# Serum neurofilament light chain levels in myasthenia gravis patients with and without symptoms

**DOI:** 10.3389/fmed.2025.1652698

**Published:** 2025-09-08

**Authors:** Jie Lv, Ruichen Liu, Zhan Sun, Jing Zhang, Yingna Zhang, Xue Zhao, Jing Liu, Xinyue Zhou, Mengdi Zhang, Qian Liu, Feng Gao

**Affiliations:** Department of Neuroimmunology, Henan Institute of Medical and Pharmaceutical Sciences, Zhengzhou University, Zhengzhou, China

**Keywords:** myasthenia gravis, neurofilament light chain (NFL), biomarker, SiMoA assay, late-onset MG

## Abstract

This study aimed to investigate serum neurofilament light chain (sNFL) levels in patients with myasthenia gravis (MG) and explore its potential as a biomarker for disease stratification. A total of 60 MG patients and 29 normal controls (NCs) were enrolled, with no significant differences in age or gender between the two groups. MG patients were stratified by MGFA classification, QMG scores, antibody status, phenotypic subtypes, onset age, and gender. Results showed that MG patients had significantly higher sNFL levels (median: 12.7 pg./mL) compared to NCs (median: 9.1 pg./mL; *p* = 0.0176). Subgroup analyses revealed that sNFL levels in MGFA-II patients (median: 13.1 pg./mL) were significantly elevated compared to NCs (*p* = 0.0437), with no statistical difference in MGFA-I. Patients with QMG scores 7–15 (median: 13.4 pg./mL) had higher sNFL levels than those with scores 0–6 (*p* = 0.0207) and showed significant differences from NCs (*p* = 0.0023). Late-onset MG (LOMG) patients (median: 13.4 pg./mL) had higher sNFL levels than early-onset cases (*p* = 0.0368), and age was mildly correlated with sNFL in MG (*p* = 0.0477). ROC analysis showed moderate diagnostic performance of sNFL for distinguishing LOMG vs. NCs (>50 years) was 0.9464 (specificity 89.29%, sensitivity 90%), and for female MG vs. female NCs was 0.8091. In conclusion, sNFL levels are elevated in MG patients, particularly in severe and late-onset cases, suggesting its potential as a biomarker for disease stratification and severity assessment.

## 1 Introduction

Myasthenia gravis (MG) is an autoimmune disease that affects the neuromuscular junction (NMJ) through specific autoantibodies (1),divided into ocular (OMG) and generalized forms (GMG) (2). “Myasthenia Gravis Foundation of America (MGFA) classifies MG into five grades to quantify severity: Grade I (ocular symptoms only); Grade IIa (mild generalized weakness, primarily affecting limbs); Grade IIb (moderate generalized weakness with bulbar involvement); Grade III (severe generalized weakness, potentially life-threatening); Grade IV (severe crisis requiring intubation); and Grade V (myasthenic crisis with respiratory failure) (3). The features discussed in this study (e.g., bulbar weakness, respiratory involvement) are most prominent in Grades IIb-V, with Grade V specifically characterized by respiratory failure due to neuromuscular junction dysfunction.” 80% of MG patients have anti-AChR antibodies, 50% of AChR-seronegative patients have anti-MuSK antibodies, and 20–30% of AChR-positive patients have anti-Titin antibodies, which are key in MG’s development (4, 5). In 100 MG patients, the detection rate of Titin antibodies was 41% (6), they are also important roles involved in the pathogenesis of MG. MG patients were classified into early-onset MG (EOMG, age at onset < 50 years) and late-onset MG (LOMG, age at onset ≥ 50 years). OMG is more common in patients with EOMG, while GMG is more common in patients with LOMG (7). The treatment effectiveness of patients with EOMG is significantly higher than that of patients with LOMG. The importance of timely diagnosis of myasthenia gravis is that early diagnosis allows for prompt initiation of treatment, slowing the progression of the disease and reducing the risk of myasthenia crisis (8). To sum up, EOMG and LOMG are different in many ways, and timely diagnosis is of great significance for the treatment and prognosis of patients.

Neurofilament proteins (NF) are important components of the neuronal cytoskeleton (9). Among neurofilament proteins, neurofilament light chain (NFL) has the smallest molecular weight (68 kDa), enabling it to more readily diffuse into cerebrospinal fluid and blood via tissue fluid during axonal injury or neurodegeneration. Neurofilaments comprise three subtypes: light (NFL), medium (NFM, 150 kDa), and heavy (NFH, 200 kDa) chains (10, 11). As the most abundant and soluble neurofilament subunit, NFL is significantly upregulated in release under pathological conditions, resulting in detectable blood concentrations. In contrast, NFM and NFH, with larger molecular weights and more complex structures, are less released into the blood and present at lower levels (12, 13). Normally, NFL is stably expressed and distributed in neurons (14), when neurons are damaged or diseased, their cell membranes lose integrity, causing NFL to leak from the neurons into the bloodstream via the blood–brain barrier (15). Neurogenic changes were regularly found in the muscles of patients with myasthenia, even without muscular atrophy (16). In MG, serum neurofilament light chain (sNFL) may reflect the degree of neuromuscular junction damage and neuronal stress, particularly in subgroups where traditional biomarkers like anti-acetylcholine receptor (AChR) and anti-muscle-specific kinase (MuSK) antibodies may be less informative (17). Basic research has shown that preserving neuromuscular junctions and modulating certain signaling pathways are crucial in age-related and other forms of muscle atrophy (18). Therefore, changes in sNFL levels are considered to be closely related to the degree of nervous system injury and are expected to become a potential biomarker reflecting the pathological process of nervous system diseases (19). In various nervous system diseases, such as Alzheimer’s disease (AD), Parkinson’s disease (PD), multiple sclerosis (MS) (20). NFL is involved in immune regulation and various autoimmune diseases, but its role in MG is still unclear. SNFL level is a reliable marker of neuronal damage (21).

Against this backdrop, it is rational to hypothesize that NFL levels may be elevated in MG and that this measurement could be beneficial for disease monitoring. The primary aim of this study was to compare plasma NFL levels between age, gender, ocular and generalized forms, antibody types (AchR, Musk, Titin), MGFA classification (Type I, IIa, IIb), and QMG scores, and controls. This study aims to investigate the expression levels of sNFL in MG patients and evaluate its potential as a biomarker for disease monitoring and pathophysiological understanding.

## 2 Materials and methods

### 2.1 Study population

We recruited 34 patients diagnosed with myasthenia gravis from May 2021 to September 2023 in ZMB and collected their clinical information and serum. All patients were diagnosed based on clinical symptoms, electrophysiological studies (including single-fiber EMG in cases of diagnostic uncertainty), and response to acetylcholinesterase inhibitors refers to objective improvement in clinical symptoms (records were taken every 10 min after injection, with continuous recording for 60 min). The relative score, calculated using the formula based on the absolute score of the single most significantly improved item at the time of maximum improvement, was used as the judgment value for the test result. A relative score < 25% was considered negative, 25–60% as suspiciously positive, and >60% as positive (22), including symptom classification at onset as ocular and generalized, antibody types (AChR, MuSK), MGFA classification (Type I, IIa, IIb), and QMG scores, were recorded. MGFA I-IIb patients were prioritized due to their higher representation in our cohort; severe grades (III-V) were excluded due to small sample size (*n* = 4, <10% of total). The control group was expanded to include 29 healthy individuals matched for age and potential comorbidities such as diabetes. Exclusion criteria for both groups included other autoimmune diseases, nervous system infectious diseases, malignant tumors, severe liver and kidney insufficiency, and recent use of drugs that may affect nerve function (23). Excess serum samples were collected after routine diagnostic procedures and stored at −80 °C. Neurofilament protein light chain levels in all sera were tested.

### 2.2 sNfL measurement

Serum samples were collected from patients with MG and controls, serum samples were collected within 2 h of venous blood draw, clotted at room temperature (22–25 °C) for 30 min, then centrifuged at 3,000 × g for 10 min at 4 °C to separate serum. Aliquots (500 μL each) were immediately stored at −80 °C without delay, with no more than two freeze–thaw cycles before analysis. Freezer temperatures were monitored daily to ensure stability between −78 °C and −82 °C. SNfL concentrations were measured using the single-molecule array (SiMoA) assay on the HD-1 analyzer (Quanterix, Lexington, MA, USA), following the manufacturer’s protocol with the two-step assay dilution 2.0 scheme of the NF-Light Advantage kit; monoclonal anti-NfL antibodies and calibrators were used by UmanDiagnostics (Umeå, Sweden).

### 2.3 Statistical analysis

Statistical analysis was conducted using SPSS 26.0 and GraphPad Prism 8. Statistical significance was set at two-tailed (*p* < 0.05), with adjustments for multiple comparisons applied as appropriate. Serum neurofilament light chain (sNfL) values were expressed as medians with interquartile ranges (IQRs), confirmed by Shapiro–Wilk tests. For group comparisons: two-group analyses utilized the non-parametric Mann–Whitney U test, given the skewed distribution of sNfL data. Multiple-group comparisons were performed using the Kruskal-Wallis test, followed by Dunn’s post-hoc test with Bonferroni correction to reduce Type I error risk. Outliers were identified using the IQR method (values >1.5 × IQR above the 75th percentile or below the 25th percentile) and excluded only after sensitivity analyses confirmed their influence on model fit. Linear, quadratic, and exponential regression models were tested to explore association shapes, with the optimal model selected based on the highest coefficient of determination (R^2^) and lowest Akaike Information Criterion (AIC). Diagnostic performance of sNfL was evaluated using receiver operating characteristic (ROC) curves, with area under the curve (AUC) and 95% confidence intervals (CIs) calculated to quantify discriminative ability. Cutoff values were optimized via Youden’s index (sensitivity + specificity − 1) to balance accuracy. Decision curve analysis (DCA) was additionally performed to assess clinical utility by comparing net benefit across threshold probabilities.

## 3 Results

### 3.1 Demographics and clinical characteristics of MG patients and healthy controls

The baseline of MG patients and controls is summarized in Table 1. A total of 60 MG patients and 29 normal controls (NCs) patients were included. There were no significant differences in age and gender between the MG and control groups. There were 28 OMG and 32 GMG cases in the MG group. AChR-Ab positive in 33 cases and MuSK-Ab positive in 17 cases. QMG scores were 0–6 in 28 MG patients and 7–15 in 30 MG patients. Other MG patients were divided into early-onset MG (EOMG, onset age < 50 years) and late-onset MG (LOMG, onset age ≥ 50 years). According to MGFA classification, the distribution ratio of I, IIa and IIb types was 32:12:12.

**Table 1:** Baseline cohort characteristics of MG patients and NCs participants.

Variables	MG patients (*n* = 60)	NCs (*n* = 12)	t/χ2	*p*-value
Sex, female, *n* (%)	30 (50%)	14 (48.3%)	0.1525	0.89
OMG: GMG, *n*	28:32	_/	_/	_/
MGFA at enrollment				
I: IIa: IIb, *n*	32:12:12	_/	_/	_/
AChR-Abs(+), *n* (%)	33 (55%)	_/	/	/
MuSK-Abs(+), *n* (%)	17 (28.3%)	_/	_/	_/
EOMG (<50) LOMG (>50)	30 (50%) 30 (50%)	_/	_/	_/
QMGs				
0–6, *n* (%)	28 (46.7%)	_/	_/	_/
7–15, *n* (%)	30 (50%)	_/	_/	_/

The Chi-squared test was used to compare the difference of gender between two groups. The unpaired t-test was used to compare the difference of ages between two groups. *n* = number of patients; *p* < 0.05 was considered significant. AChR-Abs, anti-acetylcholine receptor antibodies; MuSK-Abs, muscle-specific receptor tyrosine kinase antibodies; Titin, titan protein; EOMG, early-onset myasthenia gravis; LOMG, late-onset myasthenia gravis; GMG, generalized myasthenia gravis; OMG, ocular myasthenia gravis; NCs, normal controls; MG, myasthenia gravis; MGFA, myasthenia gravis foundation of America; *n*, numbers; QMGs, quantitative myasthenia gravis scores; SD, standard deviation.

### 3.2 Elevated serum neurofilament protein light chain levels in patients

**Figure 1:**
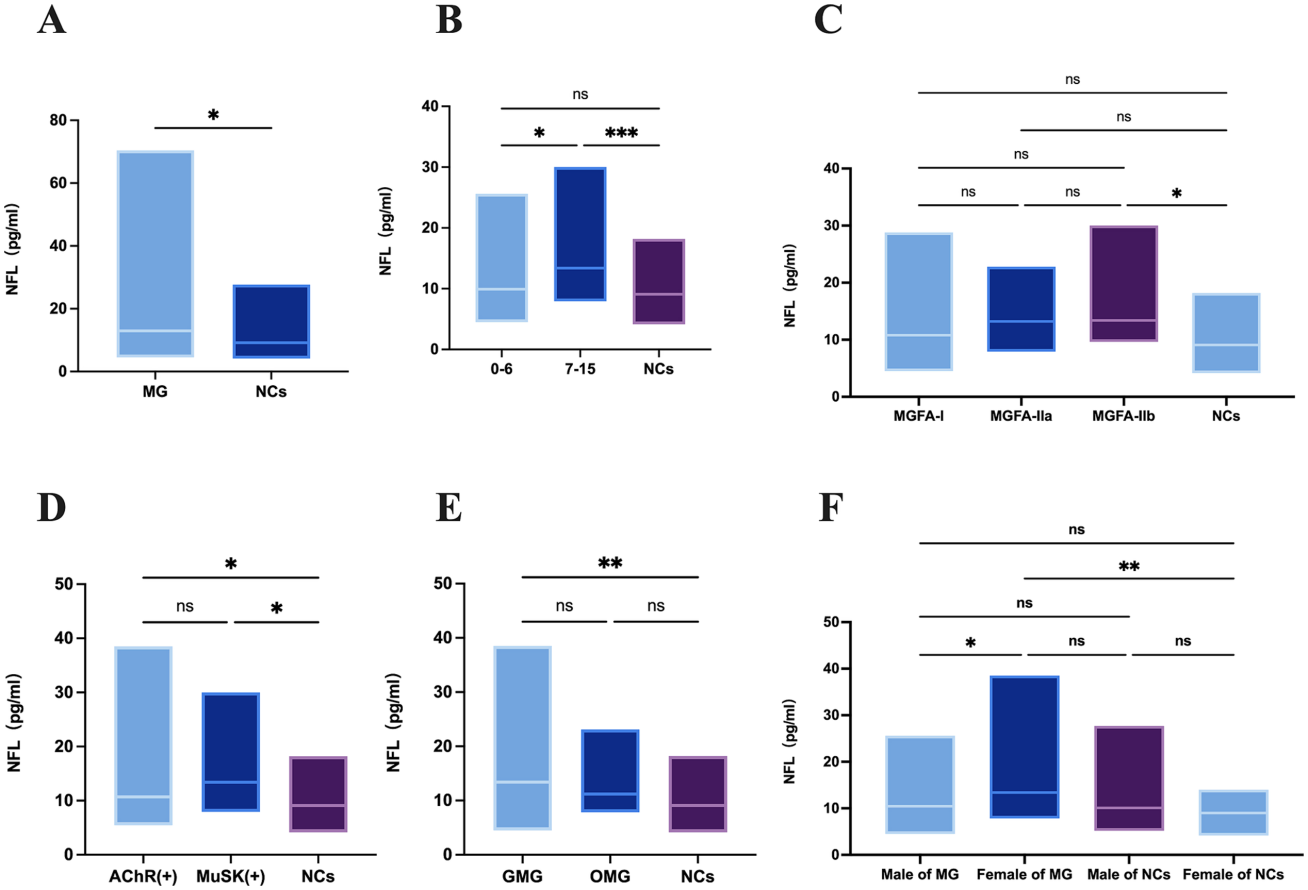
**(A)** Studies on the levels of sNFL in MG patients (*n* = 60 patients) and NCs (*n* = 29 patients). sNFL, serum neurofilament light chain; MG, myasthenia gravis; LOMG, NCs, normal controls. **(B)** Studies on the levels of sNFL in MG patients with different types of QMG scores (NCs: *n* = 26 patients; 0–6: *n* = 28 patients; 7–15: *n* = 30 patients). QMGs, quantitative myasthenia gravis scores. **(C)** Studies on the levels of sNFL in MG patients with different types of MGFA (NCs: *n* = 29 patients; MGFA-I: *n* = 32 patients; MGFA-IIa: *n* = 12 patients; MGFA-IIb: *n* = 12 patients). MGFA, myasthenia gravis foundation of America. **(D)** Studies on the levels of neurofilament light chain in MG patients with different types of antibodies (NCs: *n* = 29 patients; Anti-AChR-positive patients: *n* = 33 patients; Anti-MuSK-positive patients: *n* = 17 patients). AChR(+), acetylcholine receptor antibody positive MG-patients, MuSK(+), muscle specific positive MG patients. **(E)** Studies on the levels of neurofilament light chain in GMG (*n* = 32patients) and OMG (*n* = 28 patients). GMG, generalized myasthenia gravis; OMG, ocular myasthenia gravis. **(F)** Studies on the levels of neurofilament light chain in female (*n* = 30 patients) and male (*n* = 30 patients) MG patients. sNFL levels in female of NCs (*n* = 14) and male of NCs (*n* = 15). ^*^p < 0.05, ^**^p < 0.01, ^***^p < 0.001.

In this study, the serum neurofilament protein light chain levels between myasthenia gravis (MG) patients and normal controls (NCs) were thoroughly investigated and verified. The results demonstrated that MG patients had significantly higher levels of serum neurofilament protein light chain, reaching median 12.7 (IQR = 9.8;mean±SD: 16.0 ± 8.0) pg./ml, in contrast to the normal control group (IQR = 5.0; median 9.1, mean ± SD: 9.9 ± 3.5) pg./ml (*p* = 0.002) (Figure 1A).

Based on the Quantitative Myasthenia Gravis (QMG) scores, MG patients were divided into two groups with scores ranging from 0 to 6 and 7–15. The serum neurofilament protein light chain levels for QMG scores of 0–6 and 7–15 were median 6.9 (IQR = 7.3; mean±SD:11.5 ± 5.5) pg./ml and median 13.4 (IQR = 11.1; mean±SD:15.4 ± 5.5) pg./ml, respectively, (Figure 1B). The group of QMG scores of 7–15 had significant differences from the normal control group (*p* = 0.0007). However, there was significant difference between the serum neurofilament protein light chain levels of patients with QMG scores of 0–6 and 7–15 (*p* = 0.03).

Subsequently, we analyzed the MGFA subgroups of MG. The serum neurofilament protein light chain levels in MGFA-I was median 10.8 (IQR = 10.6;mean±SD: 12.5 ± 5.7) pg./ml. And in MGFA-IIa and MGFA-IIb were median 13.2 (IQR = 9.9; mean ± SD: 12.7 ± 6.0) pg./ml and median 13.4 (IQR = 10.6; mean±SD:15.5 ± 5.69) pg./ml, respectively. Although sNFL levels in both MGFA-I, MGFA-IIa and MGFA-IIb subgroups were higher than those in NCs, only the MGFA-IIb subgroup showed a statistically significant difference compared to NCs (*p* = 0.0101) (Figure 1C). And no difference was observed between MGFA − IIa and MGFA − IIb patients (*p* = 0.6184).

We also compared different antibody-positive subgroups of MG. The serum neurofilament protein light chain levels in AChR-Abs(+) and MuSK-Abs(+)were median 10.7 (IQR = 12.6; mean ± SD:13.4 ± 7.8) pg./ml, and median 13.4 (IQR = 10.5; mean ± SD:15.0 ± 6.1) pg./ml respectively (Figure 1D). There were no significant differences among these two different antibody-positive subgroups (*p* = 0.6,998). But there was significant difference between AChR-Abs(+) in MG and NCs (*p* = 0.0490). While, there was significant difference between MuSK-Abs(+) in MG and NCs (*p* = 0.0164).

Furthermore, potential differences within different phenotypic subgroups of MG were further explored. When comparing ocular myasthenia gravis (OMG) and generalized myasthenia gravis (GMG) patients, we found that the serum neurofilament protein light chain levels in OMG were median 13.4 (IQR = 12.1;mean ± SD:15.5 ± 8.9) pg./ml and in GMG were median 11.2; (IQR = 12.1;mean ± SD:13.0 ± 4.7) pg./ml (Figure 1E). Both subgroups had levels higher than those of the NCs. Intriguingly, there was no significant difference (*p* = 0.2654)in the serum neurofilament protein light chain levels between OMG and GMG patients. But there was significant difference between GMG in MG and NCs (*p* = 0.0030).

When comparing male and female MG patients, as well as their corresponding normal groups. The serum neurofilament protein light chain levels in male and female MG patients were median 10.5 (IQR = 7.6;mean±SD:11.7 ± 5.0) pg./ml and median 13.4 (IQR = 11.8; mean ± SD:16.7 ± 7.6) pg./ml respectively, with significant difference between them (*p* = 0.0311) (Figure 1F). In the normal control groups, the serum neurofilament protein light chain levels in male and female NCs were median 10.1 (IQR = 8.8;mean±SD = 11.5 ± 6.6) pg./ml and median 9 (IQR = 3.6;mean±SD: 9.1 ± 2.6) pg./ml respectively, with no significant difference between them (*p* = 0.5721). Nevertheless, it was notable that the serum neurofilament protein light chain levels in female MG patients had significance in normal female controls (*p* = 0.0032), and the sNFL chain levels in male MG patients also were no significantly higher than those in normal male controls (*p* = 0.7044).

**Figure 2:**
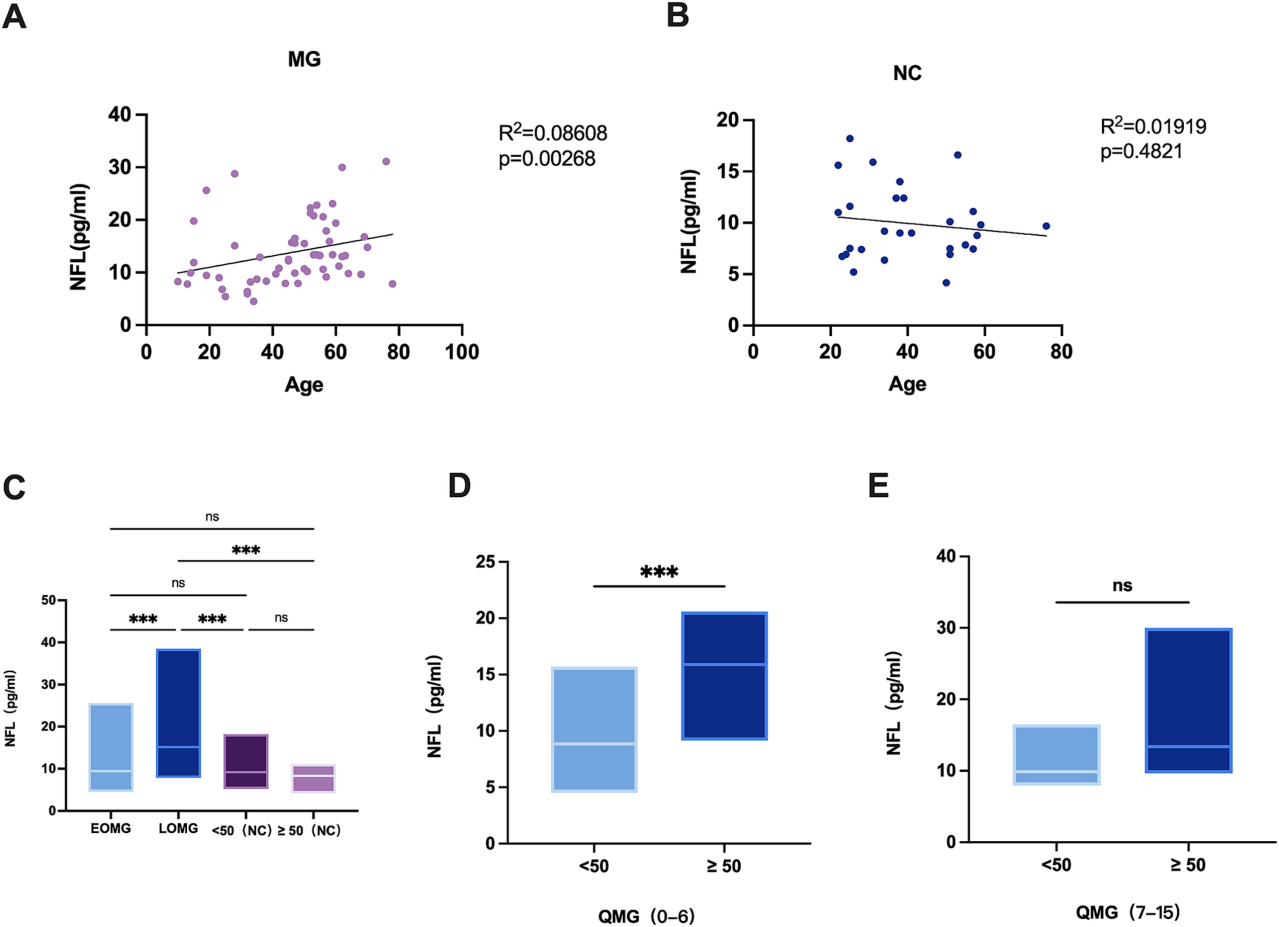
**(A)** Correlation of age with sNFL levels in MG patients (*n* = 60). **(B)** Correlation of age with sNFL levels in NCs (*n* = 29). **(C)** sNFL levels in EOMG (*n* = 30), LOMG (*n* = 30), and NCs (*n* = 29). EOMG, early-onset myasthenia gravis; LOMG, late-onset myasthenia gravis. **(D)** sNFL levels between MG patients aged < 50 years and those aged > 50 years in the quantitative myasthenia gravis (QMG) score range of 0–6; **(E)** sNFL levels between MG patients aged < 50 years and those aged > 50 years in the QMG score range of 7–15. ^*^p < 0.05, ^**^p < 0.001, ^**^p < 0.0001.

Lastly, we compared early-onset myasthenia gravis (EOMG) and late-onset myasthenia gravis (LOMG). In MG patients, the correlation analysis between age and sNFL levels showed an R^2^ of 0.08608 (*p* = 0.0268) (Figure 2A), while in NCs, the analysis revealed an R^2^ of 0.01919 (*p* = 0.4821) (Figure 2B) We noted that the sNFL chain levels in EOMG were median 9.44 (IQR = 7.3;mean±SD:11.3 ± 4.9) pg./ml and in LOMG were median 15.5 (IQR = 11.9;mean±SD:17.2 ± 7.9) pg./ml (Figure 2C). Although EOMG showed no significant difference from the normal control group, a significant difference was identified between EOMG and LOMG (*p* = 0.0368). While, the sNFL chain levels in NCs (<50 years) were median 9.2 (IQR = 7.1;mean±SD: 10.2 ± 4.5) pg./ml and in NCs (≥50 years) were median 8.3 (IQR = 2.7;mean±SD: 8.3 ± 2.1) pg./ml (Figure 2C). Further subgroup analysis stratified by QMG scores (a measure of disease severity)showed: in the QMG score range of 0–6, patients aged ≥50 years had a median sNFL level of 15.9 pg./mL (interquartile range (IQR = 6.2) pg./ml) with a mean ± standard deviation of (15.6 ± 4.6) pg./ml, which was significantly higher than that in patients aged <50 years (median 8.9 pg./mL, interquartile range (IQR = 5.0) pg./ml, mean ± standard deviation (9.1 ± 2.9) pg./ml) ((*p* = 0.0007)) (Figure 2D). In the QMG score range of 7–15, patients aged ≥50 years had a median sNFL level of 9.98 pg./mL (interquartile range (IQR = 7.7) pg./ml) with a mean ± standard deviation of (11.7 ± 5.5) pg./ml. Although this was higher than that in patients aged <50 years (median 13.4 pg./mL, interquartile range (IQR = 10.6) pg./ml, mean ± standard deviation (14.8 ± 6.1) pg./ml), the difference did not reach statistical significance ((*p* = 0.0781)) (Figure 2E). These results suggest that age itself may be an independent factor contributing to elevated sNFL levels, even when MG severity is consistent.

### 3.3 ROC curves of serum neurofilament light chain protein in different MG groupings and NCs for differential diagnosis

#### 3.3.1 ROC curve of serum neurofilament light chain protein in MG and NCs for differential diagnosis

**Figure 3:**
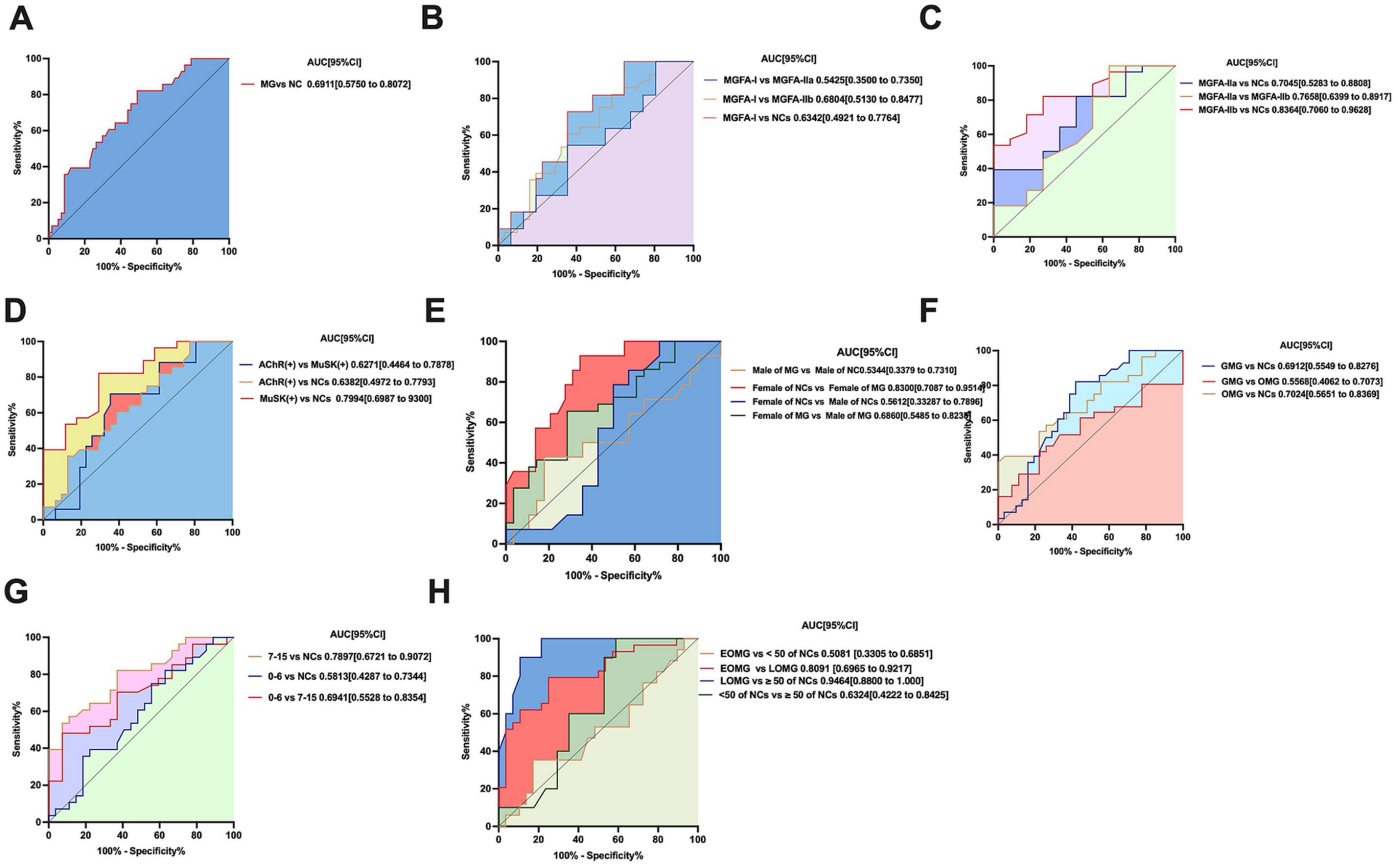
**(A)** ROC curve of neurofilament light chain distinguishing MG (*n* = 60 patients) from controls (*n* = 29 participants). *p* < 0.05, statistically significant. **(B,C)** Neurofilament light chain distinguishing MGFA-I (*n* = 32patients), MGFA-IIa (*n* = 12 patients), MGFA-IIb (*n* = 12 patients) and from controls (*n* = 29 participants) ROC curve; MGFA-I (*n* = 32 patients) vs. MGFA-IIa (*n* = 12 patients) ROC curve. MGFA-I (*n* = 32 patients) vs. MGFA-IIb (*n* = 12 patients) ROC curve; MGFA-IIa (*n* = 12 patients) vs. MGFA-IIb (*n* = 12 patients) ROC curve. MGFA-I (*n* = 32 patients) vs. MGFA-IIb (*n* = 12 patients) ROC curve. **(D)** sNFL distinguishing AChR-Ab (*n* = 33 patients), MuSK-Ab (*n* = 17 patients), from controls NCs (*n* = 29 participants) ROC curve; neurofilament light chain distinguishing AChR-Ab (*n* = 33 patients), MuSK-Ab (*n* = 17 patients) ROC curve. **(E)** Neurofilament light chain distinguishing female (*n* = 30 patients) and male (*n* = 30patients) MG patients ROC curve; female of NCs (*n* = 14) and female of MG patients (*n* = 30) ROC curve; male (*n* = 15 patients) and male of MG patients (*n* = 30) ROC curve. **(F)** Neurofilament light chain distinguishing OMG (*n* = 28 patients), GMG (*n* = 32patients) from controls (*n* = 29participants) ROC curve; OMG (*n* = 28patients) vs. GMG (*n* = 32 patients) ROC curve, statistically significant. **(G)** Neurofilament light chain distinguishing QMG scores of 0–6 (*n* = 28 patients), QMG scores of 7–15 (*n* = 30 patients) from controls (*n* = 29 participants) ROC curve; QMG scores of 0–6 (*n* = 28 patients), QMG scores of 7–15 (*n* = 30 patients) ROC curve, statistically significant. **(H)** Neurofilament light chain distinguishing LOMG (*n* = 30patients), EOMG (*n* = 30 patients) from controls (*n* = 18 participants) ROC curve; LOMG (*n* = 30 patients), EOMG (*n* = 11 patients) ROC curve. AUC, area under the curve; CI, confidence interval.

The ROC curve and values for neurofilament light chain and its combinations are shown in Figure 3A. The area under the curve (AUC) for MG and NCs was 0.6911, with a specificity of 50.88% and a sensitivity of 82.14%.

#### 3.3.2 ROC curve of serum neurofilament light chain protein in different MGFA subtypes and NCs for differential diagnosis

In the validation phase, we performed receiver operating characteristic (ROC) curve analysis to evaluate the diagnostic performance of serum neurofilament light chain (sNFL) and its combined indicators in differentiating myasthenia gravis (MG) patients classified as MGFA-I, MGFA-IIa, and MGFA-IIb. The ROC curves and corresponding performance metrics for sNFL and its combinations are presented in Figure 3B.

Specifically, when distinguishing MGFA-I from MGFA-IIa, MGFA-IIb, and normal controls (NCs), the areas under the curve (AUC) were 0.5425, 0.6804, and 0.6342, respectively. For these comparisons, the specificities were 55.88, 64.52, and 64.52%, with corresponding sensitivities of 82.14, 72.73, and 60.71% (Figure 3B).

In the differentiation of MGFA-IIa from MGFA-IIb, the AUC was 0.6405, with a specificity of 45.45% and a sensitivity of 72.73%.

Additionally, when distinguishing NCs from MGFA-IIa and MGFA-IIb, the AUC values were 0.7045 and 0.8344, respectively. For NCs vs. MGFA-IIa, the specificity was 81.82% and sensitivity was 39.29%; for NCs vs. MGFA-IIb, the specificity was 72.73% and sensitivity was 82.14% (Figure 3C).

#### 3.3.3 ROC curve of serum neurofilament light chain protein in different antibody subtypes and NCs for differential diagnosis

The ROC curve and values for neurofilament light chain and its combinations about different antibody-positive subgroups of MG are shown in Figure 3D. The AUC for AChR-Ab and MuSK-Ab with NCs were 0.6382 and 0.7994, respectively, with specificities of 22.58 and 64.71%, and sensitivities of 100 and 82.14%. The AUC for AChR-Ab vs. MuSK-Ab was 0.6271, with a specificity of 64.52% and a sensitivity of 70.59%.

#### 3.3.4 ROC curve of serum neurofilament light chain protein in male and female MG patients and NCs for differential diagnosis

The ROC curves and corresponding performance metrics for neurofilament light chain (sNFL) and its combined indicators are presented in Figure 3E. For the differentiation of male myasthenia gravis (MG) patients from female MG patients, the area under the curve (AUC) was 0.6860, with a specificity of 71.43% and a sensitivity of 65.52%. In the comparison of male normal controls (NCs) vs. female NCs, the AUC was 0.5612, accompanied by a specificity of 42.86% and a sensitivity of 85.71%. When distinguishing male MG patients from normal male controls, the AUC was 0.5344, with a specificity of 78.57% and a sensitivity of 42.86%. Notably, for the differentiation of female MG patients from normal female controls, the AUC reached 0.8300, with a specificity of 65.52% and a sensitivity of 92.86%.

#### 3.3.5 ROC curve of serum neurofilament light chain protein in phenotypic subtypes and NCs for differential diagnosis

The ROC curve and values for neurofilament light chain and its combinations different antibody-positive subgroups of MG are shown in Figure 3F. The AUC for OMG, GMG with NCs were 0.7024 and 0.6912, respectively, with specificities of 96.3 and 54.84%, and sensitivities of 39.29 and 82.14%. The AUC for OMG vs. GMG was 0.5568, with a specificity of 77.78% and a sensitivity of 41.94%.

#### 3.3.6 ROC curve of serum neurofilament light chain protein in different QMG scores and NCs for differential diagnosis

ROC curve analysis further revealed that the diagnostic performance of QMG scores in distinguishing between the 0–6 and 7–15 severity levels was relatively low (Figure 3G).

Specifically, when differentiating QMG scores of 0–6 from normal controls (NCs), the area under the curve (AUC) was 0.5813, with a specificity of 44.44% and a sensitivity of 75%. For QMG scores of 7–15 vs. NCs, the AUC was 0.7897, accompanied by a higher specificity of 92.59% and a sensitivity of 53.57%. In the direct comparison between QMG scores of 0–6 and 7–15, the AUC was 0.6941, with a specificity of 92.59% and a sensitivity of 48.15%.

#### 3.3.7 ROC curve of serum neurofilament light chain protein in LOMG and EOMG and NCs for differential diagnosis

The ROC curves and corresponding performance metrics for neurofilament light chain (sNFL) and its combined indicators in differentiating early-onset myasthenia gravis (EOMG) from late-onset myasthenia gravis (LOMG) are presented in Figure 3H. For the differentiation of LOMG from EOMG, the area under the curve (AUC) was 0.8091, with a specificity of 75% and a sensitivity of 79.31%. In the comparison of normal controls (NCs) aged < 50 years vs. NCs aged ≥ 50 years, the AUC was 0.6324, accompanied by a specificity of 41.18% and a sensitivity of 100%. Notably, when distinguishing LOMG from NCs aged > 50 years, the AUC reached 0.9464, with a high specificity of 89.29% and a sensitivity of 90%. In contrast, for the differentiation of EOMG from NCs aged < 50 years, the AUC was 0.5081, with a specificity of 82.76% and a sensitivity of 35.29%.

## 4 Discussion

Myasthenia gravis (MG) is a heterogeneous autoimmune disorder characterized by neuromuscular junction dysfunction, with clinical variability across subtypes, ages, and severity levels. Neurofilament light chain (NfL), a sensitive marker of neuronal injury, has emerged as a potential biomarker in various neurodegenerative and neuroinflammatory conditions, such as Alzheimer’s disease (AD) (24), Parkinson’s disease (PD) (25), multiple sclerosis (MS) (26). In severe viral infections like COVID-19 and herpes zoster, without signs of CNS involvement, sNfL levels may rise due to neuroinflammatory or direct neuronal injury (27, 28). NFL is involved in immune regulation, various autoimmune diseases and viral infections, but its role in MG is still unclear. This study investigated serum NfL (sNfL) levels in MG patients and healthy controls, aiming to clarify its association with disease subtypes, severity, and clinical characteristics.

Our primary finding is that sNFL levels are significantly elevated in MG patients compared to normal controls (NCs), with a median of 12.7 pg./mL in MG vs. 9.1 pg./mL in NCs (*p* = 0.002). This aligns with the hypothesis that neuromuscular junction damage and neuronal stress in MG may trigger NFL release into the bloodstream. Neurofilaments, particularly NfL, are released upon axonal injury or cytoskeletal disruption, and their detection in serum reflects ongoing pathological processes in the nervous system (10). In MG, where autoantibodies disrupt neuromuscular transmission and may induce secondary neuronal stress, elevated sNFL could serve as a proxy for the extent of tissue damage beyond clinical symptoms alone.

In the study by Hviid CVB (29), the non-parametric reference intervals for sNFL were determined as 2.8–9.7 ng/L for ages 18–40, 4.6–21.4 ng/L for 41–65 years, and 7.5–53.8 ng/L for over 65 years. Simrén J’s study established age-partitioned reference limits based on a strong relationship between age and plasma neurofilament light (30), with upper 95th percentile values of 7 pg./mL for 5–17 years, 10 pg./mL for 18–50 years, 15 pg./mL for 51–60 years, 20 pg./mL for 61–70 years, and 35 pg./mL for 70 + years. Subgroup analyses revealed critical insights into the relationship between sNFL and MG phenotypes. Late-onset MG (LOMG) patients had significantly higher sNFL levels (median 15.5 pg./mL) than early-onset MG (EOMG) patients (median 9.44 pg./mL, *p* = 0.0368), while no such age-related difference was observed in NCs. This aligns with our correlation analysis showing a weak but significant association between age and sNFL in MG (R^2^ = 0.08608, *p* = 0.0268) but not in controls (R^2^ = 0.01919, *p* = 0.4821). Furthermore, stratified analysis by QMG scores (a measure of severity) demonstrated that even among patients with matched disease severity, older MG patients (≥50 years) had higher sNFL levels, particularly in the mild subgroup (QMG 0–6, *p* = 0.0007). These results suggest that age itself may be an independent driver of sNfL elevation in MG, potentially due to age-related vulnerability of neurons to autoimmune-mediated stress or cumulative damage over time (18). The significant difference in sNFL levels between EOMG and LOMG patients may suggest that the age of onset affects the degree of nerve injury and sNFL release. This is in accordance that EOMG and LOMG are different in many ways, including treatment response and disease progression (7).

In MG, sNFL may reflect the degree of neuromuscular junction damage, as suggested by the high AUC values in certain subgroups. In comparison, NFL’s role in other autoimmune diseases like systemic lupus erythematosus may involve direct interactions with immune cells (31). The research value of NFL in MG lies in its role as a bridge between neural damage and immune dysregulation, providing critical insights into the disease’s complex pathology, optimizing clinical monitoring, and exploring neuroprotective therapeutic strategies.

Gender-specific differences were also notable: female MG patients had significantly higher sNFL levels than female NCs (*p* = 0.0032), with a high AUC of 0.8300 for distinguishing female MG from controls, whereas male MG patients showed no such difference. This gender disparity may reflect underlying differences in disease pathophysiology, as female MG patients often exhibit distinct autoantibody profiles and disease courses (7). The strong diagnostic performance of sNFL in female patients highlights its potential as a gender-specific biomarker, though further research is needed to explore the biological basis of this difference.

In terms of disease severity, sNfL levels correlated with QMG scores, with the moderate subgroup (QMG 7–15) showing higher levels than the mild subgroup (*p* = 0.03) and significant elevation compared to NCs (*p* = 0.0007). This suggests sNfL may track with disease activity, though the lack of significance in MGFA IIa vs. IIb subgroups (*p* = 0.6184) could be attributed to small sample sizes (IIa = 12, IIb = 12). Similarly, while MGFA IIb patients had numerically higher sNFL than MGFA I patients, only MGFA IIb showed significant elevation compared to NCs (*p* = 0.0101), indicating sNFL may better distinguish severe from mild or non-MG states rather than subtle severity gradations. Notably, the mechanism behind sNFL elevation in MG requires careful consideration: MG primarily impairs NMJ function through autoantibody-mediated disruption of acetylcholine signaling, rather than directly inducing Wallerian degeneration, which primarily affects the axonal trunk of central or peripheral nerves. Following injury, the distal segment of the axon undergoes degenerative changes such as disintegration and fragmentation, leading to massive release of neurofilaments (including NFL) into bodily fluids. In this process, this results in significantly elevated NFL levels, making it a classical biomarker of substantial axonal damage (13, 32). NMJ functional abnormalities mainly disrupt neural signal transmission, while the axonal trunk remains structurally intact. Thus, isolated NMJ impairment rarely increases NFL release. However, chronic NMJ dysfunction may trigger secondary neuronal stress, such as impaired axonal transport or cytoskeletal remodeling, leading to sustained NFL release (33, 34).

Diagnostic performance analyses reinforced sNFL’s utility in specific contexts. The highest AUC was observed for distinguishing LOMG from age-matched NCs (≥50 years, AUC = 0.9464), with high specificity (89.29%) and sensitivity (90%), highlighting its potential to aid in diagnosing older patients where clinical presentation may overlap with other age-related neuromuscular disorders. Additionally, sNFL performed well in differentiating MGFA IIb from NCs (AUC = 0.8344) and female MG from female controls (AUC = 0.8300), supporting its role as a complementary tool to traditional biomarkers like AChR or MuSK antibodies.

Notably, sNFL did not differ between AChR-positive and MuSK-positive subgroups, suggesting it reflects a common pathway of neuronal stress rather than antibody-specific mechanisms. And in another study sNFl levels were also higher in patients with MG compared to controls, but sNFl levels were highest in anti-AChR-Abs positive patients, followed by anti-MuSK-Abs positive, antiLRP4-Abs positive, and seronegative patients (35). This may be related to ethnic, regional, and individual differences in the included patients. However, the significant elevation of sNfL in both antibody subgroups compared to NCs (AChR: *p* = 0.0490; MuSK: *p* = 0.0164) indicates its potential to monitor disease activity regardless of antibody type.

This study has several limitations that should be acknowledged. First, the single-center design and small sample size may introduce selection bias, particularly in subgroup analyses (e.g., MGFA IIa/IIb with n = 12 and 12, respectively). The exclusion of severe MG grades (III–V) further limits the generalizability of our findings to the full spectrum of MG severity. Larger, multi-center studies are needed to validate these results. Second, the cross-sectional design precludes conclusions about sNFL’s utility in tracking disease progression or treatment response; longitudinal studies with larger cohorts are required to explore its prognostic value. Additionally, we only assessed serum sNFL levels, without corresponding cerebrospinal fluid measurements, which might more directly reflect central nervous system involvement (36). Third, the study lacked comparisons with other neuroimmune diseases, leaving unanswered whether sNFL acts as a broad-spectrum marker of neuroimmune pathology or a MG-specific indicator. Finally, the specific source and mechanisms of sNFL elevation in MG remain unclear. Future longitudinal studies with larger cohorts are needed to validate sNFL as a prognostic marker and explore its response to treatment.

In conclusion, sNFL levels are elevated in MG patients, suggesting its potential as a biomarker for disease stratification and severity assessment, with particular utility in LOMG, female patients, and moderate-severity disease. Its association with age, gender, and severity highlights its potential to enhance phenotypic characterization and monitor underlying pathological processes. As a readily measurable serum marker, sNFL could complement existing clinical and serological tools, the improving diagnosis and management of MG.

## Data Availability

The raw data supporting the conclusions of this article will be made available by the authors, without undue reservation.
